# Utility of echocardiographic parameters in predicting cardiac immune-related adverse events in Japanese patients undergoing immune checkpoint inhibitor therapy

**DOI:** 10.1007/s12574-025-00698-8

**Published:** 2025-07-25

**Authors:** Junya Yamaguchi, Tetsuji Morishita, Hiroyasu Uzui, Kanae Hasegawa, Hiroyuki Ikeda, Hiroshi Tada

**Affiliations:** 1 Department of Cardiovascular Medicine, Faculty of Medical Sciences, University of Fukui, 23-3 Matsuokashimoaizuki, Eiheiji-Cho, Yoshida-Gun, Fukui, 910-1193 Japan; 2 Department of Internal Medicine, Matsunami General Hospital, Gifu, 501-6062 Japan; 3 Department of Clinical Nursing, Faculty of Medical Sciences, University of Fukui, Fukui, Japan

**Keywords:** Immune checkpoint inhibitors, Cardiac immune-related adverse event

## Abstract

**Background:**

Immune checkpoint inhibitors (ICIs) have transformed cancer treatment but are associated with immune-related adverse events, including cardiac immune-related adverse events (cardiac irAEs). Early identification of patients at risk for cardiac irAEs is crucial, and echocardiographic parameters may serve as valuable predictors. This study aimed to evaluate the predictive utility of echocardiographic indices in assessing cardiac irAE risk in Japanese patients receiving ICI therapy.

**Methods:**

This retrospective study analyzed patients treated with ICIs at the University of Fukui Hospital between November 2015 and October 2018. Cardiac irAEs were defined according to the ESC guidelines.

**Results:**

Among 73 patients, six (8%) developed cardiac irAEs, with no fatalities. Echocardiographic assessment before ICI initiation revealed that patients who later developed cardiac irAEs had significantly lower ejection fractions (EFs) (*p* < 0.05). Receiver operating characteristic analysis demonstrated that left ventricular end-systolic diameters (LVDs) (area under the curve: AUC = 0.660) and EFs (AUC = 0.797) had moderate predictive value for cardiac irAEs. Kaplan–Meier analysis showed a higher probability of cardiac irAEs in patients with LVDs and EFs beyond specific thresholds (*p* < 0.01). Poisson regression analysis indicated a decreasing risk of cardiac irAEs over time after ICI initiation.

**Conclusion:**

Baseline echocardiographic parameters, particularly LVDs and EFs, can be useful predictors of cardiac irAEs in patients receiving ICI therapy. Early echocardiographic evaluation may facilitate risk stratification and improve monitoring strategies for cardiac irAEs***.***

**Clinical trial registration number:**

UMIN000023840.

**Supplementary Information:**

The online version contains supplementary material available at 10.1007/s12574-025-00698-8.

## Introduction

Immune checkpoint inhibitors (ICIs) have shown revolutionary therapeutic effects through a mechanism different from that of conventional anticancer drugs but have been reported to cause severe side effects in various organs of the body [1, 2]. Cardiovascular immune-related adverse events (irAEs), such as myocarditis, have a significant impact on life outcomes [3, 4].

The frequency of adverse cardiac events is generally low (< 1%), but the fatality rate is considerably high [5]. The clinical presentations of cardiac irAEs are likely to have a spectrum of mild-to-severe disease and lack specific features [6]. These presentations encompass a myriad of symptoms including arrhythmia, heart failure, chest pain, and/or myocarditis/pericarditis [7]. Thus, the recent studies focused on the early detection and diagnosis of cardiac irAE [8–10]. Currently, the identification of candidate patients with cancer who are vulnerable to cardiac irAEs remains challenging. A few pioneering studies have addressed this issue previously [11, 12].

Our understanding of cardiac irAEs is limited, and there is a need for improved recognition of the risk stratification of patients treated with ICIs. Routine cardiac screening in asymptomatic patients is still debatable [13, 14]. In addition, there is insufficient data to support the usefulness of active surveillance in patients undergoing ICI therapy [7]. Recent studies have shown that atrial fibrillation is one of the most common cardiac irAEs associated with ICI therapy [15]. Nso et al. conducted a systematic review and meta-analysis and reported the incidence and characteristics of cardiac adverse events, including atrial fibrillation, in patients receiving ICIs. There is a wide variability in the reported time-to-onset of symptoms after the administration of ICIs [1, 4, 6, 9, 16]. Effect of comorbidities, clinical characteristics, timing, and the outcomes of cardiac irAEs remain unclear.

To address this knowledge gap, we retrospectively evaluated the time-to-onset, clinical characteristics, and other predictors of cardiac irAEs, focusing on routine echocardiographic findings, in Japanese patients receiving ICI therapy.

## Patients and methods

### Study subjects and design

This is a retrospective observational study using data from the University of Fukui Hospital, of all patients receiving ICI treatment, between Nov 1, 2015, and Oct 30, 2018. The study conformed to the principles outlined in the Declaration of Helsinki. The informed consent requirement was waived because of the retrospective nature of the study. The study protocol was approved by the Ethics Committee of University of Fukui Hospital and registered with the Universal Hospital Medical Information Network Clinical Trials Registry (UMIN000023840).

All patients were treated with nivolumab, and four patients were treated with a combination of other ICIs. Cardiac irAEs were defined as myocarditis, atrial fibrillation, heart failure, pericardial effusion, tachycardia, myocardial infarction, or cardiac arrest, based on the European Society of Cardiology guideline [7]. We excluded patients undergoing hemodialysis, documented history of atrial fibrillation prior to ICI initiation, or those aged < 18 years. Eligible patients were followed-up through the last day of December 2022 via medical record review.

### Data collection and measurements

We collected data from medical chart records, including age, sex, body mass index, coexisting disease, combination anticancer therapy, medications, electrocardiograms (ECGs), chest radiograms, and transthoracic echocardiograms (TTEs). Cancer-specific covariates including cancer type, radiation therapy, and ICI type were extracted. Blood samples and medication data were extracted 1 week prior to ICI administration. ECG, TTE, and chest X-ray data were also extracted within 1 month of ICIs administration. Hypertension was defined as systolic blood pressure ≥ 140 mmHg, diastolic blood pressure ≥ 90 mmHg, or receipt of antihypertensive medication. Diabetes mellitus was defined as glycated hemoglobin ≥ 6.5% or the receipt of insulin therapy or oral medication for diabetes mellitus. Dyslipidemia was defined as a low-density lipoprotein cholesterol level > 140 mg/dL or receipt of medication for dyslipidemia. Chronic kidney disease was defined as an estimated glomerular filtration rate < 60 mL/min/1.73 m^2^. Assessment of pre-existing atrial fibrillation: we reviewed patient medical records, including past ECG reports, event recorder data, and Holter monitor recordings, to determine the presence of pre-existing atrial fibrillation. Atrial fibrillation was defined as an atrial arrhythmia lasting ≥ 30 s, captured on a 12-lead ECG, event recording, or Holter monitor recording. [17] Ischemic heart disease was defined as a history of coronary revascularization. In our cohort, all cases of ischemic heart disease were due to previous percutaneous coronary intervention (PCI), with no cases of coronary artery bypass grafting (CABG).

TTE was performed by experienced sonographers using standard echocardiographic examinations, according to the American Society of Echocardiography guidelines [18]. Sixteen Vivid E95 ultrasound systems (GE Healthcare, USA) were used, and data were analyzed using the GE EchoPAC software (v203, GE Healthcare). Left ventricular ejection fraction (EF) was measured using the modified Simpson’s method with apical two- and four-chamber views or the Teichholz method using the parasternal long-axis view. Assessment of valvular disease: valvular disease was defined as the presence of stenosis or regurgitation of any of the four heart valves (mitral, aortic, tricuspid, or pulmonic) based on standard echocardiographic criteria. The presence and severity of valvular disease were assessed by experienced sonographers using Doppler echocardiography [19, 20].

### Outcomes

We defined cardiac irAEs (such as myocardial infarction, ischemic heart disease, atrioventricular block, supraventricular and ventricular arrhythmias, sudden death, cardiomyopathy, Takotsubo-like syndrome, non-inflammatory heart failure, and myocarditis) based on the ESC guidelines [7]. The follow-up period was defined as the period of last confirmed survival in the electronic medical records. IrAEs, except cardiac irAEs, were assessed using the American Society of Clinical Oncology definition [21].

### Statistical analysis

Data are presented as frequencies and percentages for categorical variables and mean ± SD for continuously distributed variables. The Chi-square test was used to evaluate differences in categorical variables. The Mann–Whitney *U* test was used to compare continuous variables. The association between the time-to-onset of cardiac irAEs as a continuous variable and the occurrence of cardiac irAEs was plotted using restricted cubic spline (RCS) models with three knots and Poisson regression models to estimate the relative risk for time-to-first event outcomes. Receiver operating characteristic (ROC) analyses were performed to identify the optimal clinical parameter cutoff values for predicting cardiac irAEs using the Youden index. The area under the ROC curve (AUC) was also calculated. Kaplan–Meier curves were used to illustrate event-free survival, and their estimates were compared using the log-rank test. We used logistic regression analysis with RCS models with three knots to investigate the association between clinical parameters and cardiac irAEs. Statistical analyses were performed with R version 4.3.2 (www.R-project.org) using the rms package (Frank E Harrell Jr. (2019). rms: Regression Modeling Strategies. R package, version 5.1-3.1. https://CRAN.R-project.org/package=rms) and EZR (Saitama Medical Center, Jichi Medical University, Saitama, Japan, version 1.37) [22].

## Results

### Study flow

During the study period, 107 patients were treated with ICIs at our hospital. Of these, 33 patients were excluded due to missing echocardiographic data. In addition, one patient on dialysis was excluded. Therefore, 73 patients were included in this study.

### Background of study population

**Fig. 1:**
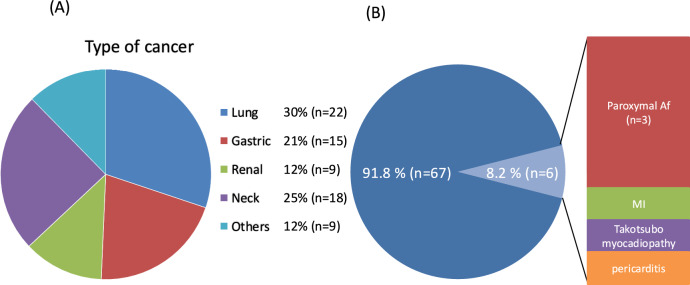
**A** Distribution of cancer type in the study population **B** Distribution of cardiac irAEs in the study population. *irAE* immune-related adverse event, *MI* myocardial infarction

The baseline characteristics of patients are presented in Table 1. The mean age of patients was 65.2 ± 11.7 years, with 55 (75%) being men. The types of malignant diseases for which ICIs were administered are shown in Fig. 1A. Of the 73 patients, cardiac irAEs occurred in 6 (8%) patients. Paroxysmal atrial fibrillation was the most common cardiac irAE resulting from ICI use (*n* = 3, Fig. 1B). Patients were evaluated between the two groups according to the presence or absence of cardiac irAEs. There were no significant differences in underlying diseases between the two groups. Significant differences were observed in medications (aspirin, and calcium channel blocker; Table 1). The cardiac irAE group had more complicated irAEs (2 cases (33%) compared to 9 cases (13%)) (Table 1).

**Table 1:** Patient characteristics

	Overall (*N* = 73)	Cardiac irAE (*n* = 6)	Without cardiac irAE (*n* = 67)	*P* value
Age, years	65.2 ± 11.7	65.0 ± 5.2	65.2 ± 12.1	0.96
Sex (male), *n* (%)	55 (75)	5 (83)	50 (75)	> 0.99
BMI, kg/m^2^	20.3 ± 3.4	20.4 ± 3.5	20.3 ± 3.5	0.94
Hypertension, *n* (%)	31 (43)	2 (33)	29 (43)	> 0.99
DM, *n* (%)	15 (21)	1 (17)	14 (21)	> 0.99
DL, *n* (%)	14 (19)	3 (50)	11 (16)	0.08
Smoking history, *n* (%)	56 (77)	4 (67)	52 (78)	0.62
Past heart disease, *n* (%)	7 (10)	1 (17)	6 (9)	0.47
Past DVT, *n* (%)	6 (8)	1 (17)	5 (8)	0.41
IHD, *n* (%)	5 (7)	0 (0)	5 (8)	> 0.99
irAE, *n* (%)	11 (15)	2 (33)	9 (13)	0.22
Medication				
ASA, *n* (%)		3 (50)	4 (6)	0.01
Statin, *n* (%)		2 (33)	8 (12)	0.19
RAS-I, *n* (%)		2 (33)	9 (15)	0.22
CCB, *n* (%)		4 (67)	15 (22)	0.04
Beta-blocker, *n* (%)		0 (0)	5 (8)	> 0.99
Diuretics, *n* (%)		0 (0)	5 (8)	> 0.99
DOAC, *n* (%)		1 (17)	3 (5)	0.30
PPI, *n* (%)		4 (67)	37 (55)	0.69
Combination therapy				
Surgical procedure, *n* (%)		2 (33)	34 (51)	0.67
Radiation, *n* (%)		1 (17)	24 (36)	0.66
Chemotherapy, *n* (%)		5 (83)	54 (81)	> 0.99
Molecular target drug, *n* (%)		2 (33)	19 (28)	> 0.99
Other ICIs, *n* (%)		0 (0)	2 (3)	> 0.99

*ASA* acetylsalicylic acid, *BMI* body mass index, *CCB* calcium channel blocker, *DL* dyslipidemia, *DM* diabetes mellitus, *DOAC* direct oral anticoagulant, *DVT* deep vein thrombosis, *ICI* immune checkpoint inhibitor, *IHD* ischemic heart disease, *irAE* immune-related adverse event, *PPI* proton pump inhibitor, *RAS-I* renin–angiotensin system inhibitor

### Association between the time-to-onset of cardiac irAE and ICI administration

**Fig. 2:**
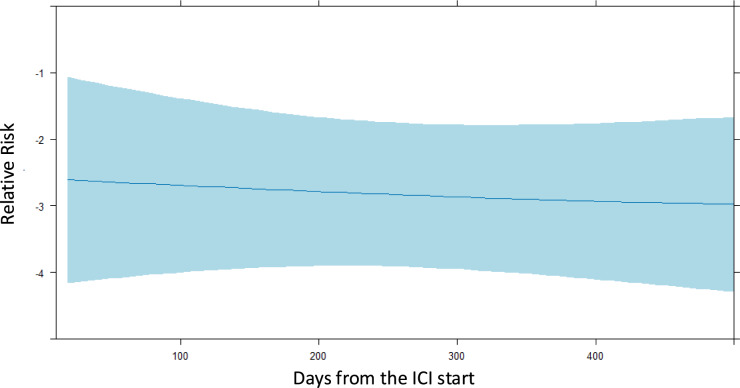
Restricted cubic spline with Poisson regression: association between cardiac irAEs and onset period. *ICI* immune checkpoint inhibitor, *irAE* immune-related adverse event

Figure 2 demonstrates the Poisson regression model with a restricted cubic spline, with the corresponding predicted cardiac irAE risk curves for the time since the administration of ICIs. The maximum follow-up period ranged from 7 to 2406 days after ICI administration, and the time of onset of cardiac irAEs ranged from 52 to 1921 days after ICI administration. The predicted risk shows a linear relationship after ICI administration.

### ECG/TTE findings and laboratory data

**Fig. 3:**
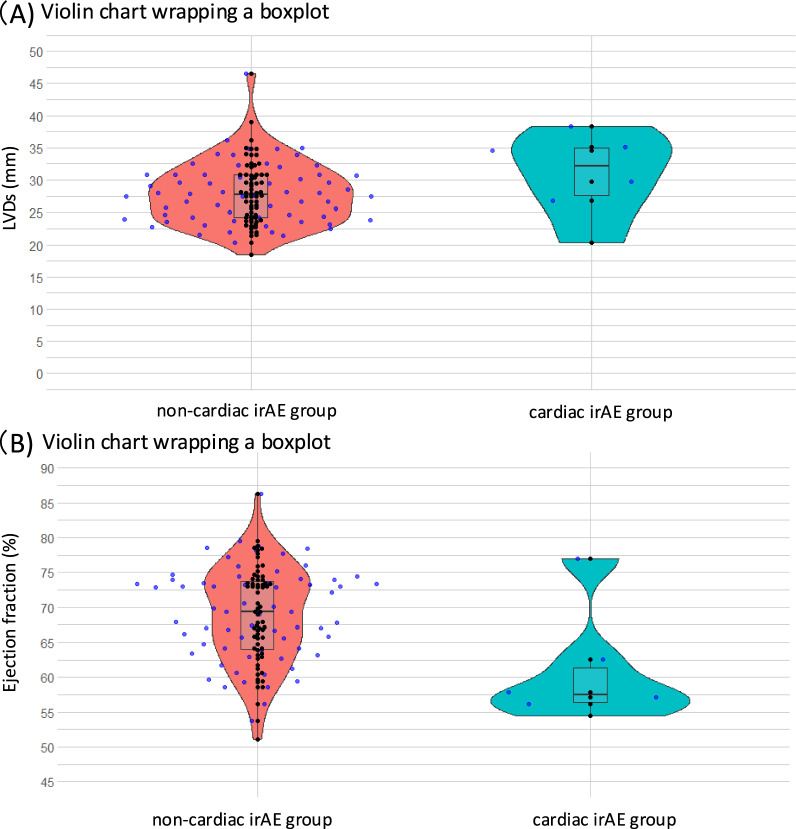
**A** Violin charts wrapping a box plot showing the LVDs values in the non-cardiac (red) and cardiac (blue) irAE groups. **B** Violin charts wrapping a box plot showing EF values in the non-cardiac (red) and cardiac (blue) irAE groups. Each point represents an individual patient and the horizontal lines indicate the medians. *EF* ejection fraction, *irAE* immune-related adverse event, *LVDs* left ventricular systolic dimension

There were no significant differences in ECG findings between the two groups (Table 2). When dichotomized with or without cardiac irAEs, hemoglobin level, platelet counts, blood urea nitrogen, serum creatinine, estimated glomerular filtration rate, albumin, and C-reactive protein values were not associated with cardiac irAEs. However, in TTE findings, while LVDs showed no significant difference (30.8 ± 6.5 vs. 28.0 ± 4.9 mm; *p* = 0.19), EFs were significantly different between the two groups (60.9 ± 8.3 vs. 68.9 ± 6.9%; *p* < 0.01) (Table 2) (Fig. 3).

**Table 2:** Laboratory, ECG, and TTE findings

	Cardiac irAE (*n* = 6)	Without cardiac irAE (*n* = 67)	*P* value
Laboratory findings			
Hemoglobin (g/dL)	11.0 ± 1.5	11.2 ± 2.1	0.77
Platelet count (10^3^/μL)	29.5 ± 5.2	26.4 ± 11.6	0.51
Blood urea nitrogen (mg/dL)	18.3 ± 16.7	16.2 ± 7.2	0.54
Serum creatinine (mg/dL)	1.04 ± 0.47	0.88 ± 0.34	0.29
Albumin (g/dL)	3.1 ± 0.4	3.5 ± 0.7	0.29
C-reactive protein (mg/dL)	2.0 ± 1.6	2.6 ± 4.2	0.75
eGFR (mL/min/1.73 m^2^)	63.4 ± 20.3	71.4 ± 24.4	0.44
ECG findings			
Pre-ECG findings			
PQ interval (msec)	151 ± 20	163 ± 24	0.23
QRS duration (msec)	89 ± 5	96 ± 19	0.43
QTc time (msec)	441 ± 19	429 ± 27	0.29
Post-ECG findings			
PQ interval (msec)	155 ± 13	163 ± 24	0.45
QRS duration (msec)	94 ± 13	101 ± 21	0.48
QTc time (msec)	438 ± 31	436 ± 53	0.93
TTE findings			
EF (%)	60.9 ± 8.3	68.9 ± 6.9	< 0.01
LVDd (mm)	45.7 ± 6.8	45.9 ± 5.7	0.9
LVDs (mm)	30.8 + 6.5	28.0 ± 4.9	0.19
LVEDV (ml)	76.9 ± 20.5	95.2 ± 25.4	0.09
LVESV (ml)	33.8 ± 13.0	31.5 ± 12.7	0.7
SV (ml)	43.1 ± 11.9	63.3 ± 15.8	< 0.01
CO (L/min)	3.4 ± 0.8	4.7 ± 1.3	0.02
E/A	0.73 ± 0.2	0.95 ± 0.4	0.2
E wave (cm/sec)	58.2 ± 8.7	70.9 ± 25.8	0.2
Deceleration time (msec)	185.5 ± 23.8	219.1 ± 57.5	0.16
E/e’ (cm/sec)	10.7 ± 2.9	12.3 ± 4.5	0.4
LAD (mm)	29.8 ± 3.5	31.9 ± 5.8	0.4
Valvular disease, *n* (%)	0 (0)	2 (3)	> 0.99

*CO* cardiac output, *ECG* electrocardiogram, *EF* ejection fraction, *eGFR* estimated glomerular filtration rate, *irAE* immune-related adverse event, *LAD* left atrial diameter, *LVEDV* left ventricular end-diastolic volume, *LVESV* left ventricular end-systolic volume, *LVDd* left ventricular diastolic dimension, *LVDs* left ventricular systolic dimension, *SV* systolic volume, *TTE* transthoracic echocardiography

### Receiver operating characteristic analysis and Kaplan–Meier analysis for cardiac irAEs

**Fig. 4:**
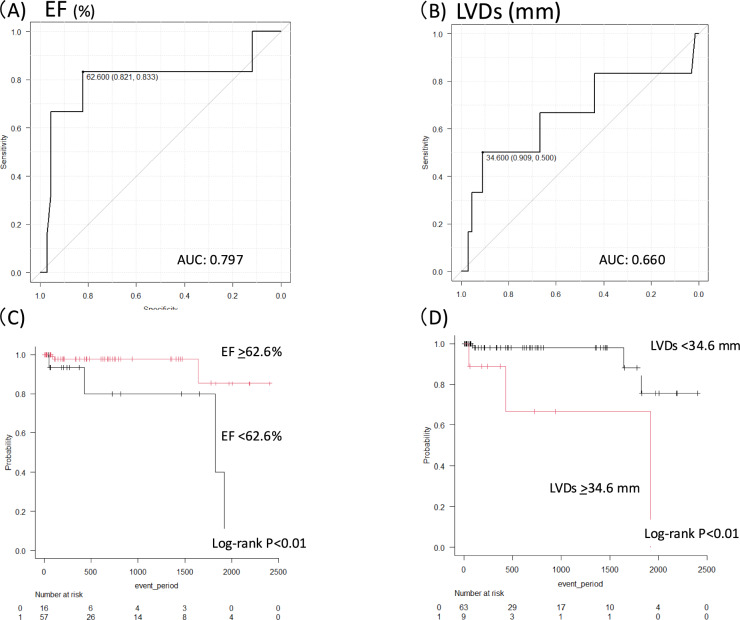
Receiver operating characteristic (ROC) curves of EF (**A**) and LVDs (**B**) for cardiac irAEs. The cutoff values (sensitivity and specificity) are shown in each figure. Kaplan–Meier curves for cardiac irAEs stratified by EF (**C**) and LVD (**D**). *AUC* area under the curve, *EF* ejection fraction, *irAE* immune-related adverse event, *LVDs* left ventricular systolic dimension

EF and left ventricular end-systolic diameter (LVDs) were analyzed by ROC curve, and cutoff value were each calculated as 62.6% (AUC: 0.80, 95% CI 0.52–1.0) (Fig. 4A) and 34.6 mm (AUC: 0.660, 95% CI 0.35–0.96) (Fig. 4B). Kaplan–Meier estimates of cardiac irAE-free survival are shown in Fig. 4C and D. The survival analysis demonstrated higher probabilities of cardiac irAEs in both the lower EF (Fig. 4C) and larger LVDs groups (Fig. 4D) (both log-rank *p* < 0.01).

### Relationship between LVDs and EF, and cardiac irAE

**Fig. 5:**
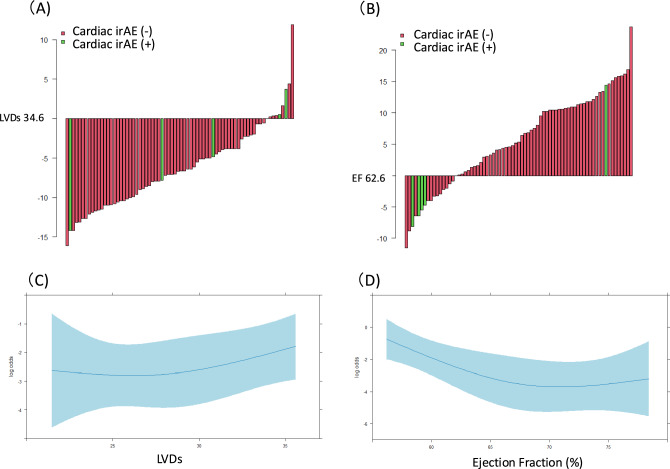
Waterfall plots showing the association of LVDs (**A**) and EF (**B**) with cardiac irAEs. LVDs and EF values are expressed as the differences from their cutoff values (34.6 mm and 62.6%, respectively) for each patient, in increasing order of values. Restricted cubic spline with logistic regression: association between cardiac irAEs and LVDs (**C**) and EF (**D**). *EF* ejection fraction, *irAE* immune-related adverse event, *LVDs* left ventricular systolic dimension

Waterfall plots of the association between LVDs, EF, and cardiac irAEs are shown in Fig. 5A and B. LVDs and EF values are expressed as the difference from each cutoff values (34.6 mm and 62.6%, respectively) for each patient in decreasing order of values. Individual waterfall plots can be created for each patient, and each feature value can be visualized as a bar graph that is either larger or smaller than the cutoff value. The waterfall plot of an individual LVD and EF value (difference from each cutoff value) ranged from − 16.1 to 11.9 mm and − 11.5 to 23.7%, respectively.

### Association between LVDs and EF values, and the incidence of cardiac irAE

Figure 5C and D depicts the logistic regression model with a restricted cubic spline and the corresponding predicted cardiac irAE risk curves for LVDs and EF values. The association between the risk of cardiac irAEs and LVDs is almost linear. Analysis of the relationship between EF and cardiac irAEs showed that the risk of cardiac irAEs decreased gradually until EF reached 65%. For patients with an EF > 65%, the curve of the risk of cardiac irAEs plateaued as the EF increased.

Post hoc multivariate analysis, adjusting for a history of ischemic heart disease, was performed to further evaluate the predictive value of echocardiographic parameters. After adjustment, LVEF remained a significant independent predictor of cardiac irAEs (*p* < 0.01). Although LVDs did not reach statistical significance in the adjusted model (*p* = 0.15), the restricted cubic spline analysis showed a similar trend to the unadjusted analysis (Supplementary Figs. 1 and 2).

## Discussion

The present study revealed the following findings: first, six patients (8%) developed cardiac irAEs in this retrospective cohort, and the risk of cardiac irAEs was time-dependent and decreased over time in the Poisson regression model with RCS. Second, significantly more patients with cardiac irAEs had lower EFs before ICI treatment than those without cardiac irAEs. Third, the ROC analysis demonstrated the moderate predictive ability of LVDs and EFs. In addition, the Kaplan–Meier analysis showed that the probability of experiencing cardiac irAEs was higher in the high LVDs and low EF groups. Lastly, there were linear and nonlinear relationships between LVDs and EF and the occurrence of cardiac irAEs in the logistic regression model with RCS.

In our study, the time-to-onset of cardiac irAEs was within 3 months of ICI initiation. The reported time for symptoms to appear after ICI administration varies widely [1, 4, 6, 9, 16]. However, most cardiac irAEs occur within 1 year, and the results of the present study support the findings of previous studies. The Poisson regression results also suggested a time-dependent decrease in the risk of developing cardiac irAEs. These results highlight the importance of close monitoring, especially immediately after initiation of ICI therapy.

Limited data is available on the predictive factors for the occurrence of subsequent cardiac irAEs with ICI therapy [11, 12]. Our study revealed that a decline in EFs and increase in LVDs on TTE at baseline surveillance were associated with future cardiac irAEs in patients receiving ICI therapy. The RCS model also demonstrated an association between LVDs and EFs and a gradual increase in the risk of cardiac irAEs. The post hoc analysis, adjusting for ischemic heart disease, further supports the utility of echocardiographic parameters, particularly LVEF, in predicting cardiac irAEs. This finding suggests that baseline cardiac function, as assessed by echocardiography, may play a crucial role in the development of cardiac irAEs, independent of pre-existing ischemic heart disease. Machine learning approach has been used previously, to predict cardiovascular events associated with ICIs [11]. This pioneering study reported that immunological factors (e.g., percentage of lymphocytes), oncological factors (e.g., low body weight), and history of cardiac disease could predict cardiac risk. In addition, a single-center observational study reported that a lower global longitudinal strain on echocardiography was strongly associated with major adverse cardiovascular events in ICI myocarditis [12]. However, imaging techniques for global longitudinal strain measurement and/or cardiac magnetic resonance imaging are less accessible than blood testing, ECG, or routine echocardiography. The identification of patients at high risk of cardiac irAEs using an ideal screening tool that is easily accessible in routine practice remains a challenge.

The observation that a significant proportion of patients with EF below the calculated cutoff value of 62.6% experienced cardiac irAEs underscores the importance of pre-treatment risk stratification. Based on our findings, we propose that patients with pre-existing echocardiographic abnormalities, including those with EF on the lower end of the normal range, may benefit from a modified follow-up strategy. This could include more frequent monitoring of ECGs to detect arrhythmias such as atrial fibrillation, as well as serial measurements of cardiac biomarkers such as troponin to identify early signs of myocardial injury. Further studies are needed to determine the optimal monitoring protocol for these high-risk patients and to evaluate the impact of such interventions on clinical outcomes.

This study has some limitations. Because this study was conducted in a single-center, tertiary referral hospital in this area, selection bias was possible. The pre-treatment cardiac workup lacked a standardized protocol, timing, and biomarkers. LVEF measurement by echocardiography is known to have inherent variability, with reported error rates of approximately 5–10%. This variability could have affected the accuracy of our LVEF measurements and the strength of the association between LVEF and cardiac irAEs. Also, LVEF was measured using two different methods (modified Simpson’s method and Teichholz method), which could have introduced additional bias. Although we attempted to minimize this bias using experienced sonographers and standardized protocols, we acknowledge that the lack of a single, consistent measurement method is a limitation of our study. In addition, this study retrieved data from daily clinical practice; thus, troponin and/or natriuretic peptide data were not fully investigated. We did not routinely measure CRP and high-sensitivity troponin I levels in patients who developed cardiac irAE. Therefore, we were unable to investigate the potential relationship between atrial inflammation and the development of atrial fibrillation in the context of ICI therapy. Future studies should consider incorporating these biomarkers to further elucidate the mechanisms underlying cardiac irAEs. Our data did not include serial measurements of echocardiographic assessments and only included cardiac surveillance data at baseline. Thus, we may have been unaware of asymptomatic cardiotoxicity in some patients.

## Conclusion

The risk of cardiac irAEs is time-dependent and decreases over time in patients receiving ICI therapy. LVDs and EF values on TTE before ICI treatment initiation are predictive of subsequent cardiac irAEs. The findings shed light on the timing of cardiac irAEs and the characteristics of susceptible cases, to assist in effective scheduling creation and early intervention in post-chemotherapy cardiac irAE monitoring in the limited resources of healthcare.

## Supplementary Information

Below is the link to the electronic supplementary material.

Supplementary file1 Supplementary Fig. 1 Restricted cubic spline with logistic regression adjusting for ischemic heart disease: association between cardiac irAEs and LVDs (TIFF 1590 KB)

Supplementary file2 Supplementary Fig. 2 Restricted cubic spline with logistic regression adjusting for ischemic heart disease: association between cardiac irAEs and EFS (TIFF 1590 KB)

## Data Availability

The data presented in this article cannot be shared publicly because of the privacy of the individuals who participated in the study. The data will be shared by the corresponding author upon reasonable request.
