# Supplementary figures and images for: Utility of echocardiographic parameters in predicting cardiac immune-related adverse events in Japanese patients undergoing immune checkpoint inhibitor therapy
Source: J Echocardiogr. 2025 Jul 25;24(1):14–23. doi: 10.1007/s12574-025-00698-8 (PMC12967468; doi:10.1007/s12574-025-00698-8)

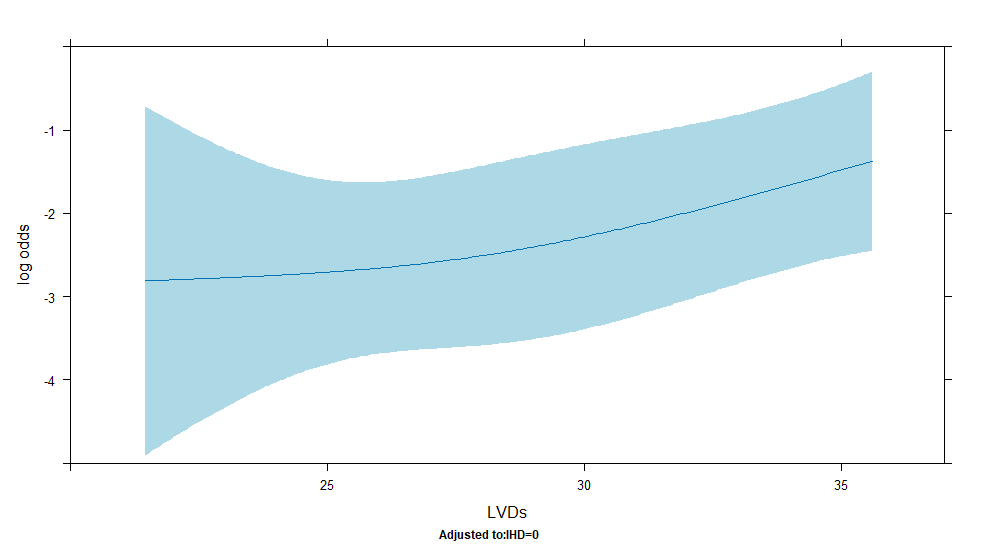

Supplement: Supplementary file 1 — Supplementary file1 Supplementary Fig. 1 Restricted cubic spline with logistic regression adjusting for ischemic heart disease: association between cardiac irAEs and LVDs (TIFF 1590 KB) [file 12574_2025_698_MOESM1_ESM.tiff]

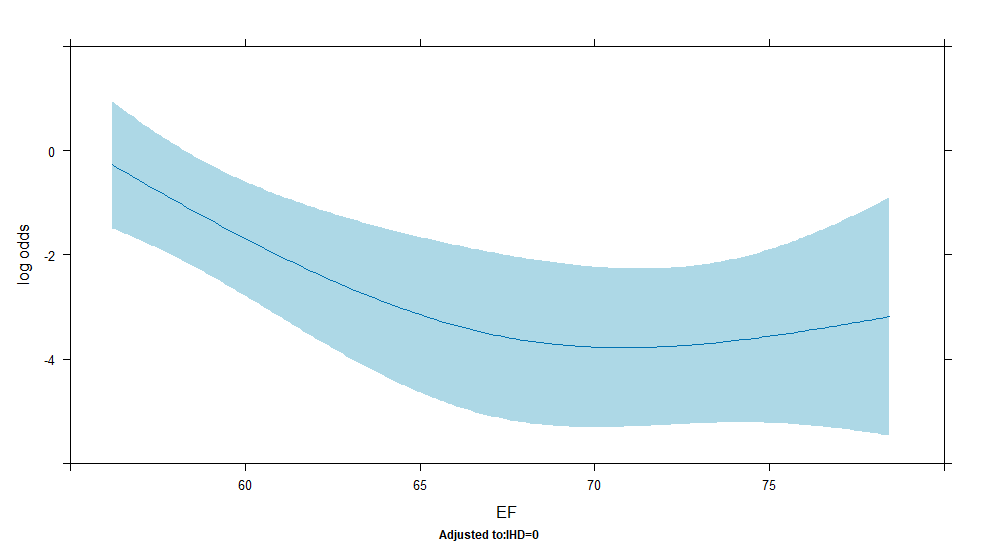

Supplement: Supplementary file 2 — Supplementary file2 Supplementary Fig. 2 Restricted cubic spline with logistic regression adjusting for ischemic heart disease: association between cardiac irAEs and EFS (TIFF 1590 KB) [file 12574_2025_698_MOESM2_ESM.tiff]
